# Discovery and Replication of Gene Influences on Brain Structure Using LASSO Regression

**DOI:** 10.3389/fnins.2012.00115

**Published:** 2012-08-06

**Authors:** Omid Kohannim, Derrek P. Hibar, Jason L. Stein, Neda Jahanshad, Xue Hua, Priya Rajagopalan, Arthur W. Toga, Clifford R. Jack, Michael W. Weiner, Greig I. de Zubicaray, Katie L. McMahon, Narelle K. Hansell, Nicholas G. Martin, Margaret J. Wright, Paul M. Thompson, The Alzheimer’s Disease Neuroimaging Initiative

**Affiliations:** ^1^Imaging Genetics Center at the Laboratory of Neuro Imaging, Department of Neurology, UCLA School of MedicineLos Angeles, CA, USA; ^2^Mayo ClinicRochester, MN, USA; ^3^Department of Radiology, UC San FranciscoSan Francisco, CA, USA; ^4^Department of Medicine, UC San FranciscoSan Francisco, CA, USA; ^5^Department of Psychiatry, UC San FranciscoSan Francisco, CA, USA; ^6^Department of Veterans Affairs Medical CenterSan Francisco, CA, USA; ^7^School of Psychology, University of QueenslandBrisbane, QLD, Australia; ^8^Center for Advanced Imaging, University of QueenslandBrisbane, QLD, Australia; ^9^Queensland Institute of Medical ResearchBrisbane, QLD, Australia

**Keywords:** neuroimaging, MRI, imaging genetics, GWAS, LASSO, *MACROD2*

## Abstract

We implemented least absolute shrinkage and selection operator (LASSO) regression to evaluate gene effects in genome-wide association studies (GWAS) of brain images, using an MRI-derived temporal lobe volume measure from 729 subjects scanned as part of the Alzheimer’s Disease Neuroimaging Initiative (ADNI). Sparse groups of SNPs in individual genes were selected by LASSO, which identifies efficient sets of variants influencing the data. These SNPs were considered jointly when assessing their association with neuroimaging measures. We discovered 22 genes that passed genome-wide significance for influencing temporal lobe volume. This was a substantially greater number of significant genes compared to those found with standard, univariate GWAS. These top genes are all expressed in the brain and include genes previously related to brain function or neuropsychiatric disorders such as *MACROD2*, *SORCS2*, *GRIN2B*, *MAGI2*, *NPAS3*, *CLSTN2*, *GABRG3*, *NRXN3*, *PRKAG2*, *GAS7*, *RBFOX1*, *ADARB2*, *CHD4*, and *CDH13*. The top genes we identified with this method also displayed significant and widespread *post hoc* effects on voxelwise, tensor-based morphometry (TBM) maps of the temporal lobes. The most significantly associated gene was an autism susceptibility gene known as *MACROD2*. We were able to successfully replicate the effect of the *MACROD2* gene in an independent cohort of 564 young, Australian healthy adult twins and siblings scanned with MRI (mean age: 23.8 ± 2.2 SD years). Our approach powerfully complements univariate techniques in detecting influences of genes on the living brain.

## Introduction

Genome-wide association studies (GWAS) offer a powerful approach to discover genes that affect individual risk for developing neurological and psychiatric disorders. Several high-density GWAS studies of Alzheimer’s disease (AD), in particular (e.g., Grupe et al., 2006), have identified genes that consistently affect AD risk across multiple populations and ethnic groups, with varying degrees of aggregated evidence. Among others, these include highly replicated risk genes such as *ApoE*,^1^ *CLU*,^2^ *PICALM*,^3^ and *CR1*^4^ (Corder et al., 1993; Harold et al., 2009; Lambert et al., 2009), as well as others supported by moderate evidence *GAB2*,^5^ *GOLM1*,^6^ *TRPC4AP*,^7^ *LRAT*,^8^ *ATXN1*,^9^ *CD33*,^10^ and *FAM113B*^11^ (Coon et al., 2007; Abraham et al., 2008; Bertram et al., 2008; Li et al., 2008; Beecham et al., 2009; Podulso et al., 2009). Most of these GWAS studies search for SNPs with alleles that are over-represented in very large samples of diseased versus matched control populations (often numbering tens of thousands of subjects). Diagnostic status, as defined in these case-control studies, is often ascertained based on a battery of cognitive tests, which are relatively remote from the molecular mechanisms of gene action in the brain. As such, studies of genetic variants that are over-represented in certain diagnostic groups often require samples of 30,000 subjects to find and replicate associations (Frayling et al., 2007).

Neuroimaging measures have been proposed as intermediate phenotypes (also known as endophenotypes) to overcome these limitations (Gottesman and Gould, 2003; Meyer-Lindenberg and Weinberger, 2006; Hall and Smoller, 2010). Several projects, such as the Alzheimer’s Disease Neuroimaging Initiative (ADNI^12^) have also sought biomarkers that are most highly associated with disease risk or with accelerated progression of the disease (Beckett et al., 2010). Imaging measures may offer several advantages for genetic analyses. As an entire 3D image of measurements is collected, rather than one single measure, the full arsenal of statistical methods developed for images – including voxel-based statistics and multivariate methods – may be used to identify spatial patterns of gene effects. Image-wide searches may also detect promising associations. Voxel-set selection may then be used to boost the power of replication attempts, by focusing on promising brain regions that may show larger effect sizes (Vounou et al., 2010, 2012). Such approaches boost statistical power by carrying only the most promising voxels into secondary analyses of independent datasets (Chen et al., 2010; Thompson et al., 2010).

Several neuroimaging measures are associated with AD (Frisoni et al., 2010; Jack et al., 2011), and some of these have already been analyzed using GWAS. Genome-wide studies of the ADNI dataset have identified new candidate AD risk genes including *TOMM40*^13^ (Potkin et al., 2009) and *GRIN2B*^14^ (Stein et al., 2010a). Traditionally, GWAS studies consider each genotype’s effect independently; they generally ignore the statistical interdependence between variants, such as their linkage disequilibrium (LD) structure, which makes certain variants more likely to be inherited together. More sophisticated GWAS approaches, using information on genes and even pathways, jointly consider groups of genetic variants that are correlated (Neale and Sham, 2004; Luo et al., 2010). Recently, neuroimaging studies have also begun to use these multi-SNP methods (Inkster et al., 2010; Hibar et al., 2011a,b; Kohannim et al., 2011; Wang et al., 2012). Here, we implement a gene-centric approach based on the least absolute shrinkage and selection operator (LASSO), to detect and model genetic influences on neuroimaging measures. LASSO is a form of regularized regression (Tibshirani, 1996), that assesses the combined effect of many correlated variables through a sparsity (or efficiency)-driven L^1^ penalty.

Here, we use the LASSO algorithm to select sparse groups of SNPs within genes. We then model the effects of the resulting SNPs jointly, to discover associations with neuroimaging measures in the ADNI dataset. Our aim was to detect more genes that influence brain structure with replication potential, as individual SNP variants have individual effect sizes that are usually hard to detect in neuroimaging datasets of the size available today, unless very large meta-analyses are performed (The ENIGMA Consortium, 2011; Stein et al., 2012). LASSO and similar penalized regression techniques have been successfully applied in the context of GWAS or candidate gene studies for selection of SNPs (Ayers and Cordell, 2010; Shi et al., 2011), detection of gene-gene interactions (D’Angelo et al., 2009; Li et al., 2011), and risk prediction from top GWAS hits (Kooperberg et al., 2010).

We hypothesized that using this approach, we would reduce the number of variants of interest in a gene to sparse sets of SNPs and thereby identify genes reliably associated with temporal lobe volume. (The method would work with other brain measures too; temporal lobe volume is just an example of specific interest, due to its role as a biomarker of neurodegenerative disease.) We tested the reliability and reproducibility of our top genetic association findings in an independent non-overlapping young adult cohort. Our goal was to see if the results could be replicated in a cohort scanned on a different continent, with a different scanner field strength, and with a roughly 50-year difference in mean age. Our motivation was to find gene effects that might persist across the human lifespan. Clearly, such a second sample presents challenges for replication, and we selected it to demonstrate the robustness of the results. We admit that this very stringent approach is only likely to find gene effects with an enduring influence throughout life, and would not serve to replicate biologically important effects present at only one part of the lifespan.

## Materials and Methods

### Subjects

Neuroimaging and genetic data were obtained from 818 subjects as part of ADNI, a 5-year study launched in 2004 by the NIH, private pharmaceutical companies, and non-profit organizations, as a public–private partnership. The goal of ADNI is to determine biological markers of Alzheimer’s disease through neuroimaging, genetics, neuropsychological tests, and other measures in order to develop and monitor new therapies, and reduce the time of clinical trials. Subjects were recruited from 58 sites across North America. The study was conducted according to the Good Clinical Practice guidelines, the Declaration of Helsinki, and U.S. 21 CFR Part 50 – Protection of Human Subjects, and Part 56 – Institutional Review Boards. Written informed consent was obtained from all participants before protocol-specific procedures were performed. All data acquired as part of this study are publicly available (see text footnote 12).

All ADNI subjects underwent thorough clinical and cognitive assessment at the time of scan acquisition to establish diagnosis. In this study, only the baseline 1.5-T scans were used (not the longitudinal follow-up data). The Mini-Mental State Exam (MMSE) was administered to provide a global measure of mental status (Cockrell and Folstein, 1988). The Clinical Dementia Rating (CDR) was used to assess dementia severity (Morris, 1993). Healthy volunteer status was determined if a subject had MMSE scores between 24 and 30 (inclusive), a CDR of 0, and was non-depressed, non-mild cognitive impairment (MCI), and non-demented. MCI diagnosis was determined if a subject had MMSE scores between 24 and 30 (inclusive), a memory complaint, objective memory loss measured by education adjusted scores on the Wechsler Memory Scale Logical Memory II, a CDR of 0.5, absence of significant levels of impairment in other cognitive domains, essentially preserved activities of daily living, and an absence of dementia. AD was diagnosed based on the National Institute of Neurological and Communicative Disorders and Stroke and the Alzheimer’s Disease and Related Disorders Association (NINCDS-ADRDA) criteria for probable AD (McKhann et al., 1984), MMSE scores between 20 and 26 (inclusive), and CDR of 0.5 or 1.0. Definitive autopsy-based diagnosis of AD was not possible.

Our ADNI dataset consisted of 729 subjects (mean age: 75.5 ± 6.8 SD years; 173 AD, 358 MCI and 198 cognitively healthy controls) with available neuroimaging, genome-wide genetic data, and other relevant covariates (age, sex, and population structure parameters derivable from the GWAS). As our replication sample, we analyzed the Brisbane healthy young adult dataset, which consists of neuroimaging and genome-wide genetic data from 564 young adult healthy twins (a mixture of monozygotic and dizygotic) and siblings of European descent (mean age: 23.8 ± 2.2 SD years; Wright and Martin, 2004). None of the subjects had a history of significant head injury, neurological or psychiatric illness, substance abuse or dependence, or had a first-degree relative with a psychiatric disorder. All subjects were screened, using a detailed neurocognitive evaluation (de Zubicaray et al., 2008) to exclude cases of pathology known to affect brain structure. Handedness was assessed based on 12 items from Annett’s Handedness Questionnaire (Annett, 1970). This sample presents some challenges for replication, due to the wide age difference (of roughly 50 years) between it and the ADNI sample. As such we were aware that genes with age-dependent effects might be found in one cohort that might not replicate in the other, for biological reasons rather than limitations in power and sample sizes.

### Neuroimaging

Structural brain MRI scans were acquired for ADNI subjects using 1.5 T MRI scanners. A subset of the ADNI subjects was also scanned at 3 T, but their scans were not used here to avoid any confounding effects of field strength. A sagittal 3D MP-RAGE sequence was used, that had been optimized for consistency across sites (Jack et al., 2008; TR/TE = 2,400/1,000 ms; flip angle = 8°; FOV = 24 cm; final reconstructed voxel resolution = 0.9375 × 0.9375 × 1.2 mm^3^). All scans were then linearly registered to the stereotaxic space defined by the International Consortium for Brain Mapping (ICBM-53; Mazziotta et al., 2001) using a 9-parameter transformation (three translations, three rotations, and three scales; Collins et al., 1994). Three-dimensional maps of regional brain volumes, computed relative to an average brain template, were generated with a method known as tensor-based morphometry (TBM), a well-established method for mapping volumetric differences in the brain (Hua et al., 2008a,b), using a minimal deformation template (MDT) from the healthy elderly group as reference. Temporal lobe volumes were obtained by integrating the Jacobian determinants of the deformation transform over the region defined as the temporal lobe on the mean anatomical template. Average temporal lobe volumes (average of lobar volumes in the left and right hemisphere) were considered as phenotypes for genome-wide association, and 3D maps were retained for use in *post hoc* analyses. The same temporal lobe volume phenotype was used in a prior study by Stein et al. (2010a).

In the Brisbane young adult cohort, all subjects were imaged on one scanner with structural whole-brain MRI at 4 T (Bruker Medspec). *T*_1_-weighted images were acquired with an inversion recovery rapid gradient echo sequence [TI/TR/TE = 700/1,500/3.35 ms; flip angle = 8° slice thickness = 0.9 mm, with a 256^3^ acquisition matrix]. All images were corrected for intensity non-uniformity (Sled et al., 1998) within an automatically delineated mask of the brain (Smith, 2002). Images were spatially normalized to the ICBM-152 template (Mazziotta et al., 2001) using a 9-parameter (global) transformation that rotated and scaled each image to minimize a normalized mutual information cost function (Jenkinson et al., 2002). Images were then resampled in the space of the template using sinc interpolation to yield 1 mm^3^ isotropic voxels. In this way, each brain was globally matched in size and mutually aligned, but local differences in shape and size remained intact. Similarly to ADNI, TBM was used to create maps of volumetric differences for each subject, using a reference MDT specially constructed for this young adult cohort.

### Genotyping

The ADNI genotyping procedures are thoroughly described in Saykin et al. (2010). Briefly, genotypes were imputed, using Mach (version 1.0)^15^ as in Stein et al. (2010b). Imputation is commonly used in genetics to infer or impute unmeasured genotypes, based on available genotypes and known patterns of correlation among markers. We used imputation in ADNI to estimate haplotype phasing and remove missing genotype calls, yielding a set of SNPs without missing data. We used the Plink software^16^ to extract SNPs that had minor allele frequencies greater than 0.1 (10%), and Hardy–Weinberg equilibrium *p*-values less than 5.7 × 10^−7^. Hardy–Weinberg equilibrium refers to the principle that frequencies of alleles, and their corresponding genotypes, are in the expected proportions, under certain assumptions about the population. We only considered alleles with a minor allele frequency > 0.1 due to the very large sample sizes needed to detect effects of less common variants (Flint et al., 2010). As in Stein et al. (2010b), we also considered only unrelated Caucasian subjects identified by self-report and verified by multidimensional scaling (MDS) analysis (Stein et al., 2010a). This was done to decrease the effects of population stratification, although we additionally included MDS parameters as covariates in this study. As detailed in Hibar et al. (2011a), we extracted and grouped all intragenic SNPs, excluding those not located in any genes. Only SNPs within transcripts (i.e., introns and exons, including untranslated regions) were included; SNPs upstream or downstream from genes were not included to avoid arbitrary window sizes. Similarly, imputation of missing genotype calls based on haplotype phasing was performed in the Brisbane young adult cohort.

### Gene-image association

#### SNP selection with the LASSO algorithm

We incorporated all genotyped SNPs in a gene into LASSO regression models and obtained coefficients for each intragenic SNP. The LASSO (Tibshirani, 1996) is a form of regularized or “penalized” regression, where L^1^ regularization is introduced into the standard multiple linear regression procedure, using a compound cost function to optimize the regression coefficients:

$$\beta^{*}=\arg\underset{\beta }{\min}{∥y-X\beta ∥}^{2}+\lambda {∥\beta ∥}_{1}$$

Here, *y* represents the vector of our neuroimaging measure co-varied for age and sex (i.e., residuals of linear regression after adjustment for the covariates), *X* is the matrix of genotypes for a single gene coded additively for the number of minor alleles (i.e., 0, 1, or 2), and β* represents the vector of fitted regression coefficients for each SNP’s effect on the neuroimaging measure. λ is a positive, weighting parameter on the L^1^ penalty, which encourages sparsity in the resulting set of fitted regression coefficients. In other words, a regression model with a smaller number of coefficients is favored, but this desirable characteristic is traded-off with the need for a model that offers a good fit to the data (which is measured by the first term above). Leave-one-out cross-validation was performed to determine the optimal penalty parameter with the mean squared error criterion. The LASSO analysis was performed gene by gene, and relatively sparse subsets of SNPs within each gene were obtained with non-zero coefficients, by fitting each LASSO model using optimal parameters. For our analyses, we used the “glmnet” package (Friedman et al., 2010) implemented in R^17^, which optimizes model fitting parameters using a coordinate descent algorithm. We performed single leave-one-out cross-validation cycles with the coordinate descent algorithm for each fold and found regularization parameters that led to the smallest average mean squared errors across all folds.

#### Multi-SNP partial *F*-tests

Subsets of SNP genotypes within genes selected by the LASSO were incorporated into partial *F*-tests, and *p*-values were obtained for their joint effect on imaging phenotypes, after adjustment for age and sex. Partial *F*-tests were performed for each gene, where the full model included SNP genotypes selected by LASSO, along with covariates, and the reduced model contained only covariates. In ADNI, population structure parameters from MDS analysis were also included as covariates. For the Brisbane twin cohort, we used a version of the efficient mixed-modeling association (EMMA; Kang et al., 2008) software, modified to allow for subject relatedness when fitting multiple regressors in the regression model. The LASSO step used above serves as a filter to yield a less noisy and more sparse set of predictors, similarly to the data-adaptive *collapsing* method used in Chen et al. (2011) and the principal component regression method used in Hibar et al. (2011a). Although leave-one-out cross-validation is used to obtain the optimal parameters for LASSO, the SNPs that remain in the optimized LASSO model are incorporated into partial *F*-tests in the same dataset.

#### Correction for multiple comparisons

In genetics, there is a notion of genome-wide significance, based on the premise that credible results should surpass a very stringent statistical threshold that accounts for the very large number of separate statistical tests performed when imaging measure is associated with a large number of different variants on the genome. To compute an appropriate threshold to determine the genome-wide significance level for our gene-centric tests, we used a Bonferroni correction: *p* = 0.05/(number of genes; i.e., 18,284), which yields a *p*-value threshold of 2.73 × 10^−6^. To perform *post hoc*, exploratory tests on the top genes, we created voxelwise statistical maps using partial *F*-tests from a multiple linear regression fitted at each voxel. To correct for multiple spatial comparisons, we used a regional False Discovery Rate (FDR) method, which is now fairly standard in neuroimaging (Langers et al., 2007).

## Results

### Gene-image association results

**Figure 1:**
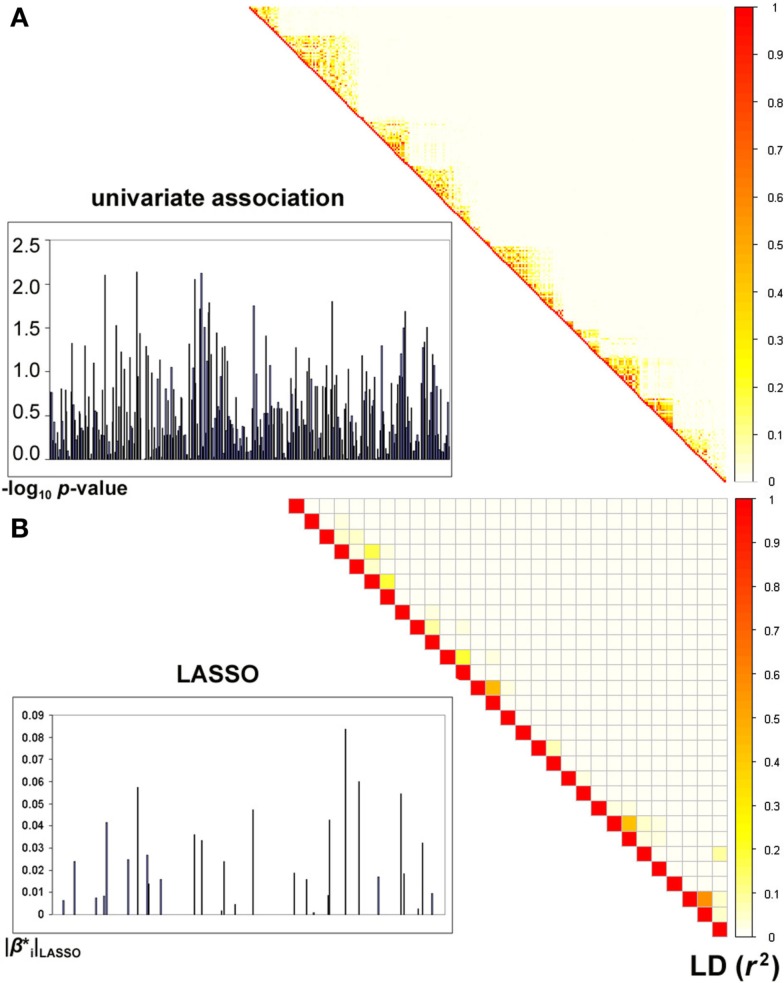
**Comparison of univariate and LASSO association tests with an imaging-derived measure of temporal lobe volume**. **(A)** *p*-values are shown, in the left panel, as −log_10_ *p* (i.e., after logarithmic transformation) for all 291 genotyped SNPs in the *MACROD2* gene, using univariate association with temporal lobe volume. The most significant SNP in this gene has a *p*-value of 7.3 × 10^−3^. A matrix representing the pairwise correlation (i.e., *r*^2^), due to linkage disequilibrium (LD), between the SNPs is displayed in the right panel. **(B)** LASSO considers all SNPs in a gene jointly and assigns sparse coefficients to the SNPs. Here, the absolute values of the coefficients $\left|\beta_{\text{i}}^{*}\right|$ are displayed for all SNPs in *MACROD2*, 29 of which attained non-zero coefficients as part of the sparse regression model fitted by the LASSO method. When considered jointly in a multiple linear regression model, the selected SNPs yield a boosted *p*-value of 7.94 × 10^−12^ for *MACROD2* (see Table 1). As in **(A)**, the correlation structure between the selected SNPs is shown, demonstrating reduced multicollinearity.

Sparse groups of SNPs within genes were selected by the LASSO algorithm and were subsequently incorporated into partial *F*-tests of association with the MRI-derived measure of temporal lobe volume (see Figure 1 for an example). We found a total of 22 genes to be genome-wide significant (after Bonferroni correction for the total number of genes assessed). Remarkably, the top gene (*MACROD2*), had a *p*-value of 7.94 × 10^−12^ (Table 1; also see later for independent replication of this top hit). We compared *F*-test *p*-values for significant genes with univariate *p*-values corresponding to single top SNPs in the same genes (similarly to Figure 2 in Hibar et al., 2011a). In the standard, univariate GWAS (Stein et al., 2010a), two genes passed genome-wide multiple comparison correction: *GRIN2B* and *NRXN3*. Here, we found the same two among our 22 significant genes, and the whole-gene *p*-values we obtained for both were “more significant” (i.e., had greater effect sizes) than their top SNP *p*-values reported in the GWAS (on the order of 10^−9^ and 10^−8^ instead of 10^−7^ and 10^−6^, respectively). The other 20 genes were undetectable with univariate GWAS as none of their SNPs attained an individual *p*-value that passed genome-wide significance. We confirmed that all top genes were expressed in the brain, using the Tissue-specific Gene Expression and Regulation (TIGER) database (Liu et al., 2008; Table 1). Although *MACROD2* and *GALNTL6* were not found in this database, their expression in the brain was evident from the GeneNote software (Shmueli et al., 2003).

**Table 1:** **Genes showing significant associations with an MRI-derived measure of temporal lobe volume**.

Gene name	Gene description	Chr.	Enr.	*N*_SNPs_	*p*-value
*MACROD2*	MACRO domain containing 2, isoform 2	20	N/A	29	7.94 × 10^−12^
*SORCS2*	Sortilin-related VPS10 domain containing receptor 2	4	2.08	29	4.87 × 10^−9^
*GRIN2B*	Glutamate receptor, ionotropic, *N*-methyl-d-aspartate 2B	12	4.60	4	7.95 × 10^−9^
*GALNTL4*	UDP-*N*-acetyl-alpha-d-galactosamine:polypeptide *N*-acetylgalactosaminyltransferase-like 4	11	1.55	12	2.39 × 10^−8^
*NRXN3*	Neurexin 3	14	2.75	5	2.84 × 10^−8^
*AK130123*	cDNA FLJ26613 fis, highly similar to serine/threonine protein phosphatase 2A, 55 kDa regulatory subunit B, alpha isoform	8	1.08	20	3.83 × 10^−8^
*MAGI2*	Membrane associated guanylate kinase, WW and PDZ domain containing 2	7	3.76	16	8.44 × 10^−8^
*NPAS3*	Neuronal PAS domain protein 3	14	3.10	7	9.06 × 10^−8^
*RBFOX1*	Ataxin-2-binding protein 1	16	4.64	3	3.24 × 10^−7^
*AY229892*	FIP1L1/PDGFRA fusion protein	4	1.65	11	3.97 × 10^−7^
*ZMAT4*	Zinc finger, matrin-type 4	8	3.76	13	6.93 × 10^−7^
*STAG3L2*	Stromal antigen 3-like 2	7	2.03	10	7.97 × 10^−7^
*GAS7*	Growth arrest-specific 7	17	2.92	9	9.60 × 10^−7^
*KIAA1217*	KIAA1217	10	0.30	13	9.65 × 10^−7^
*ADARB2*	Adenosine deaminase, RNA-specific, B2	10	3.00	17	1.01 × 10^−6^
*GABRG3*	Gamma-aminobutyric acid (GABA) A receptor, gamma 3	15	3.21	26	1.01 × 10^−6^
*CDH4*	Cadherin 4	20	0.91	22	1.12 × 10^−6^
*CLSTN2*	Calsyntenin 2	3	2.40	7	1.37 × 10^−6^
*CDH13*	Cadherin 13	16	2.08	7	1.39 × 10^−6^
*GALNTL6*	UDP-*N*-acetyl-alpha-d-galactosamine:polypeptide *N*-acetylgalactosaminyltransferase-like 6	4	N/A	5	1.80 × 10^−6^
*PRKAG2*	Protein kinase, AMP-activated, gamma 2 non-catalytic subunit	7	1.50	16	1.81 × 10^−6^
*CHODL*	Chondrolectin	21	2.04	17	2.27 × 10^−6^

*Here we show the official names, gene descriptions, corresponding chromosomes (Chr.), enrichment scores for expression of the genes in the brain (Enr.), number of selected SNPs (*N*_SNPs_), and *p*-values for genes with significant associations with temporal lobe volumes. The genes are ranked in order of significance. Here, the *p*-value threshold for significance is 2.73 × 10^−6^, which is the Bonferroni-corrected for the total number of genes, which is less strict than that for the total number of SNPs. *p*-Values correspond to *F*-tests, which assess the joint effect of a sparse subset of the genotyped single nucleotide polymorphisms (SNPs) in the respective gene, selected using the LASSO algorithm, on temporal lobe structure. N/A indicates genes are not found in this particular gene expression database, but we ensured there is brain expression for these genes using another database (see text). *N*_SNPs_ refers to the number of variants retained in the optimized LASSO models for each gene, which were then fed into partial *F*-tests. Only *GRIN2B* and *NRXN3* possessed SNPs (2 SNPs in *GRIN2B* and 1 in *NRXN3*) that passed a liberal genome-wide significance threshold of 1 × 10^−5^ in standard GWAS*.

### *Post hoc*, voxelwise analyses

To study the effects of the genes we identified in more spatial detail, we conducted *post hoc*, voxelwise analyses, where the partial *F*-test analysis is repeated at each voxel, and *p*-value maps are obtained for the gene effects, after correction for multiple comparisons across all temporal lobe voxels. We note in advance that this type of follow-up analysis does not provide any additional statistical verification of the effects, above and beyond the evidence given to the gene for association with the overall temporal lobe volume, in the LASSO regression model. The purpose was mainly to explore whether the volumetric effect could be also detected diffusely at the voxel level, and if so how widespread the effect was in the brain.

**Figure 2:**
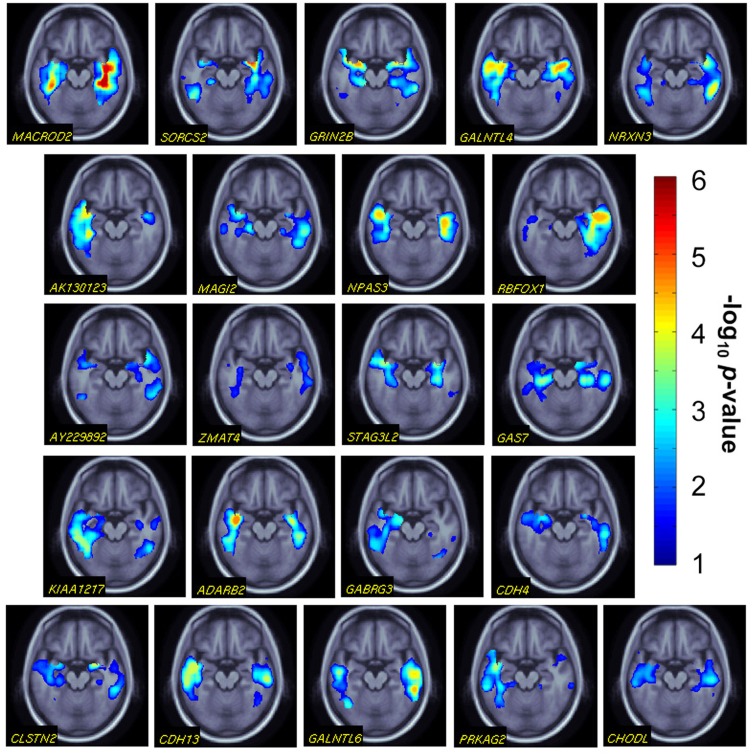
***Post hoc* gene effects on temporal lobe structure**. We conducted voxelwise associations for the 22 significant genes we identified (gene names are shown at the bottom left of the images). A representative, axial slice is shown for all gene effects. Warmer colors represent more significant effects (i.e., higher effect sizes in the analysis of overall temporal lobe volume). *p*-values are corrected for multiple comparisons within the 3D image search region, using a regional false discovery rate method.

The top genes we identified showed extensive, statistically significant effects on maps of temporal lobes’ volumetric differences (Figure 2). These are not to be considered as providing independent evidence of the effects, they simply pick up a pattern of regional effects that is likely to contribute to the aggregate effect of the gene on the temporal lobe volume.

### Replication of gene-image associations

**Figure 3:**
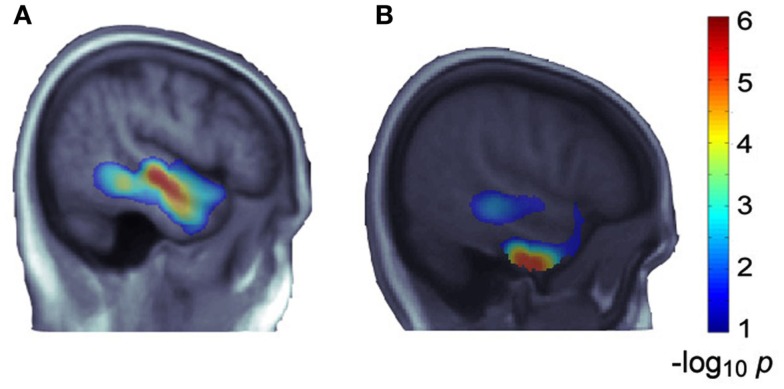
***MACROD2*’s effects in ADNI temporal lobes and partial replication in a younger cohort**. The SNPs selected from *MACROD2* in ADNI are strongly associated with mean temporal lobe volumes (*p* = 7.94 × 10^−12^), and reveal extensive and significant effects on TBM maps of temporal lobes **(A)**. We studied the joint effect of the same group of SNPs in *MACROD2* on temporal lobe maps of the Brisbane young adult dataset. The gene effect showed reproducibility, including effects at some specific locations overlapping with the ADNI findings, after correction for multiple comparisons across the temporal lobe voxels **(B)**. A sagittal slice from the left hemisphere is shown; warmer colors represent more significant associations. Both **(A,B)** are taken from the same slice (*x* = 53 mm in Montreal Neurological Institute coordinates). The slices do not appear identical due to the age range differences between the populations [e.g., atrophy in **(A)**], but almost all of the significant voxels in the more central cluster in **(B)** overlap with the significant voxels in **(A)**, implying replication.

To replicate our most significant finding (i.e., *MACROD2*) from ADNI, we explored the voxelwise effects of this same gene on temporal lobe maps from *N* = 564 healthy young adult twins and siblings. We jointly considered the same group of SNPs in the gene, selected by the LASSO method in ADNI, and studied their effect on the younger subjects’ temporal lobe maps, adjusting for sex, age, and relatedness. After correcting for multiple comparisons, *MACROD2*’s association showed partial reproducibility, as it was statistically significant across ∼15% of the temporal lobe voxels (versus ∼52% of voxels in ADNI), much of which overlapped with those in ADNI (see Figure 3).

### Cross-validation

As mentioned in the Section “Materials and Methods,” leave-one-out cross-validation was used to find optimal LASSO parameters, but no further loop of cross-validation was used for the *F*-tests. In separate analyses, we performed a nested cross-validation, where SNP selection with optimized LASSO was performed in a fifth of the dataset and the joint partial *F*-tests were then conducted in the rest of the data. This was repeated for fivefolds. The average *p*-values we obtained for our top genes with this approach did not reach genome-wide significance, mostly because the selection of SNPs with LASSO was unstable in the relatively small fractions (i.e., fifths) of the data.

We took another nested cross-validation approach, where a randomly selected half of the data was used to estimate LASSO coefficients, and the other half for partial *F*-tests. This, we presumed, might provide more balanced sample sizes for the SNP selection and for the *F*-test steps. After repeating this scheme across 10 trials, we obtained average *p*-values for our top genes. Only *GRIN2B* obtained gene-wide significance averaged across all trials (*p* = 1.47 × 10^−6^), and its association was not boosted when compared to that obtained from a univariate approach.

## Discussion

We set out to discover and replicate gene effects on brain structure using a gene-centric LASSO regression approach. The goal of the method was to sift through the vast amount of genomic data and come up with a more efficient set of variants for association testing. LASSO allowed us to select sparse subsets of SNPs among all correlated SNPs within each gene and associate them jointly in partial *F*-tests with an MRI-derived temporal lobe volume measure. Using this approach, we identified over twenty genes with significant effects on temporal lobe structure in *N* = 729 elderly subjects from the ADNI cohort – a considerably larger number of genes, when compared to a univariate approach, which considers the association of single SNPs one-by-one. In all 22 genes identified, multi-SNP *p*-values (from partial *F*-tests), using SNPs selected by LASSO, were “more significant” (i.e., lower *p*-values, greater effect sizes) than the top genotyped SNP within each corresponding gene, as computed using standard univariate GWAS. *GRIN2B* and *NRXN3*, which were identified with univariate GWAS (Stein et al., 2010a), were boosted from *p*-values on the order of 10^−7^ and 10^−6^ to 10^−9^ and 10^−8^, respectively. In addition, new genes with more significant *p*-values were discovered, whose SNPs’ individual *p*-values were too weak to pass genome-wide significance in a more standard univariate GWAS experimental design. Furthermore, *post hoc* analysis revealed widespread and significant, voxelwise influences for the top genes on TBM maps of the temporal lobes. We also replicated, at least in part, the spatial effects of our most significant finding in the *MACROD2* gene in an independent cohort of healthy, young adults.

Penalized regression techniques such as LASSO (Tibshirani, 1996), ridge regression (Hoerl, 1962), and the elastic net (Zou and Hastie, 2005) have recently been highly effective when used in GWAS. They all deal with (1) multicollinearity due to LD, (2) the large dimensionality of the genome, and (3) the problem of multiple comparisons (Malo et al., 2008; Cho et al., 2009, 2010; Lin et al., 2009; Shi et al., 2011). LASSO’s emphasis on sparsity is particularly useful in our study, as it helps point to a small set of independent variants in a given gene, which we can then incorporate into a multiple regression framework. This is similar to the approach taken by Chen et al. (2011), in the context of jointly considering rare and common variants. Here, we tested this algorithm in the context of finding genetic influences on an imaging-derived measure of temporal lobe volume. This allowed us to discover and replicate a great number of genes relative to our earlier imaging GWAS study (Stein et al., 2010a). We were also able to implicate several genes with previously identified relevance to brain disorders (see below).

In a recent study by our group, Hibar et al. (2011a) also considered gene-based associations with brain images with a new method based on principal component regression, which associates genes with images by capturing most of the variation among intragenic SNPs. Our approach complements this method, as it instead emphasizes sparsity of the model based on the available SNP data for each gene. In the case of principal components regression, a rather different line of analysis is taken in which the covariance in a set of *N* genotyped SNPs is analyzed to produce a reduced set of *k* (<*N*) predictors, that encode some of the genetic variance, but are more efficient than the original set. That method can also produce overall *p*-values for a specific gene to quantify their effects on brain structure. However, the results of principal components regression are less readily ascribed to any specific sets of SNPs on the genome.

Although the *p*-values we obtained for the two genes discovered in univariate GWAS were more significant than the univariate *p*-values of their top SNPs, this need not be the case for every gene. As also discussed in Hibar et al. (2011a), there are cases where a univariate test for the top SNP in a gene offers more power to detect an effect than a multivariate *F*-test for the whole gene. *ADAMTS2*, for instance, is a gene that contains a SNP with the lowest univariate *p*-value (rs12513486, *p* = 2.23 × 10^−5^) in our dataset just below the significance threshold considered in Stein et al. (2010a). With our *F*-test approach following LASSO regression, the gene actually obtained a weaker association (*p* = 4.83 × 10^−5^). Thus, our approach complements univariate GWAS, but does not always boost detection power by including multiple loci.

Several of the genes we identified have been well studied in the context of psychiatric and neurological disorders, including Alzheimer’s disease. Our most significant gene, *MACROD2*, which we also replicated in a new cohort, was recently discovered in the context of autism spectrum disorder (ASD), as the gene containing the top SNP (*p* < 5 × 10^−8^) in a GWAS of 1,558 families of whom some members had been diagnosed with ASD (Anney et al., 2010). The investigators of that study reported that although the precise function of this gene is mostly unknown, it is involved in several biological functions and the region comprising their top SNP may regulate *PLD2*, a gene coding for a member of a protein family with significant implications for ASD. *MACROD2* has also been associated with schizophrenia, as the gene corresponding to a rare, copy number variant in a linkage analysis (Xu et al., 2009). The same gene has also been associated with MRI-defined brain infarcts, as the gene comprising the top SNP (*p* < 5 × 10^−7^) in a meta-analysis GWAS of >9,000 mostly white, European subjects from the Cohorts for Heart and Aging Research in Genomic Epidemiology (CHARGE) consortium with an average age of 69.7 years (Debette et al., 2010). Interestingly, this study also found a suggestive association for a SNP in *GALNTL4*, another top gene in our list.

We additionally found boosted associations for another gene, *GRIN2B*, coding for a subunit of an *N*-methyl-d-aspartate (NMDA)-type glutamate receptor, and *NRXN3*, coding for a neurexin, important for synaptic function, both of which were previously identified in the same dataset with standard GWAS (Stein et al., 2010a).

We also found significant associations for *SORCS2* and *MAGI2*, which are in the AlzGene^18^ database of genes that show promising associations with the risk for developing AD based on the literature (Rogaeva et al., 2007; Potkin et al., 2009). Additionally, *NPAS3* has been linked to schizophrenia and bipolar disorder (Pickard et al., 2009), *CLSTN2* has been associated with memory performance (Papassotiropoulos et al., 2006) and with Alzheimer’s disease (Liu et al., 2007), and *RBFOX1* (*A2BP1*) has been very recently discovered as a splicing regulator of neuronal excitation and calcium homeostasis in the brain (Gehman et al., 2011). *RBFOX1* has been associated with autism, among other brain disorders (Martin et al., 2007), and an *RBFOX1* variant has interestingly been detected in another sparse regression study in ADNI (Vounou et al., 2012).

The discovery and replication populations in this study are quite different: the ADNI cohort consists of elderly subjects within the spectrum of Alzheimer’s disease and the Brisbane cohort consists of healthy young adults. Though the cohorts share a Caucasian background, which minimizes genetic heterogeneity, the age, and health differences between them implies that their brain structure may be influenced by genetic risks and mechanisms that partially overlap but also partially differ. By choosing cohorts that differ in age, we can find polymorphisms that are of enduring relevance over the lifespan, but may be less able to confirm gene effects that only matter in old age. Unfortunately, another elderly cohort with GWAS and MRI scans was not available to us at this time; we requested published GWAS data from other elderly cohorts whose MRI scans we have already analyzed, but our request was declined. However, as discussed in Stein et al. (2011) who discovered and replicated genetic variants on MRI-derived caudate volume in the same two cohorts, there may be genes with persistent effects over the human lifespan, so any such replication may be even stronger than one observed between more similar populations. In addition, though the genes we identified here may not all be AD genes, the effect of a well-known AD risk conferring polymorphism in the clusterin gene (*CLU*), and that of *GAB2*, another AD gene, have been replicated as showing associations with brain structure in the same young adult cohort (Braskie et al., 2011; Hibar et al., 2012). In other words, we knew in advance that genes associated with brain structure in the elderly may also exert detectable effects in scans from younger people. This was also the case for some SNP effects that were reliably replicated by two very large GWAS consortia analyzing brain scans from cohorts across the lifespan (Stein et al., 2012) or in elderly cohorts (Bis et al., 2012). In this study, we were similarly interested to see if our top gene’s effects on temporal lobe structure would replicate in the young, adult cohort, suggesting a more lasting influence on brain structure across a person’s lifetime.

In our study, SNPs were coded additively, i.e., using a value of 0, 1, or 2 for the number of minor alleles. This coding makes the assumption that all SNPs considered in the analysis exert their effects in an additive fashion, as opposed to alternative models such as recessive or dominant. This is certainly not the case for all SNPs, and the assumption likely affects the statistical power of our results, since greatest power is obtained when the true model of a causal allele is implemented (Lettre et al., 2007). This is a potential limitation of our study. We chose the additive model as previous genome-wide analyses of the same dataset relevant to our work implement the same allelic coding and were successful in finding genetic associations that were later replicated (Stein et al., 2010a; Hibar et al., 2011a). Furthermore, the additive model is the most commonly used association model. It is the model assumed in heritability calculations and has been argued to be closest to actual risk models for complex traits, such as our quantitative imaging-based measure (Balding, 2006).

A possible limitation of our results is that we do not implement a nested cross-validation approach, in which SNPs selected from LASSO regression would have been included in *F*-tests in non-overlapping subjects. Our implementation of LASSO here, however, fits into a filtering rather than predictive framework and similar data-adaptive filtering followed by *F*-tests in the same dataset has been done in previous work (Chen et al., 2011; Hibar et al., 2011a). This approach is potentially unfair, as fitting in LASSO is followed by another fitting with multiple linear regression (*F*-tests) in the same dataset, whereas fitting is only performed once in a univariate scheme. As GWAS are sensitive to sample size, a nested cross-validation scheme, though more robust, would most likely yield no significant results. We observed this power limitation, as we attempted nested approaches with varying numbers of folds, and were unable to obtain boosted gene-based associations. This may change in the future, as larger datasets become more widely available. Our use of a replication cohort, however, does add credibility to the top result, as the same set of SNPs selected in the discovery sample show significant, spatial effects on brain scans from a completely independent (non-overlapping) group of subjects scanned with a different scanner on a different continent. Another limitation of our approach is that we focus on genes, but exclude promoter and intergenic SNPs. This has the drawback of missing potentially important regulatory elements in the genome.

Our work has several possible future directions, biologically and methodologically. Further investigation is needed to clarify the roles of the genes we identified. We did create voxelwise maps for the top genes, but one could also use a more computationally demanding imaging GWAS approach by re-running the gene-centric, LASSO at each voxel in the brain (Stein et al., 2010b; Vounou et al., 2010; Hibar et al., 2011a), instead of running it on summary measures derived from the images. Sparse coding, used here to reduce the dimensionality of the genomic data, could also be used to zero in on the most promising voxels in the images, leading to a set of phenotypes in the images that show greatest association. Vounou et al. (2010, 2012), in particular, have proposed a general “reduced rank” method that distils a set of genes and brain measures from regions of interest into a more manageable set for assessing associations. Other approaches for dimension reduction, within both the image and the genome, involve variants of independent components analysis (Liu et al., 2009). In a recent advance, Chiang et al. (2011b) proposed to use genetic correlations to identify pairs of voxels in an image with common genetic determination, rather than simply phenotypic correlation. This could be more promising in principle than using phenotypic covariance, as it seems voxel sets are influenced by common (partially overlapping) sets of genes. By clustering these voxels into regions of interest, Chiang et al. (2011b) were able to boost power to detect genome-wide associations in a large DTI study. Clearly, the promise of multivariate methods for imaging genomics is high. Several variants of linear regression, penalized regression, and machine learning are now being adapted to handle images, with the main goal of boosting power and reducing the very large samples typically considered necessary for replicable findings in genetics (The ENIGMA Consortium, 2011).

In addition to GWAS, an alternative more hypothesis-driven approach is to use candidate gene studies to study the influence of genetic variants on brain structure. These have recently been successful in implicating genes as associated with brain white matter integrity measures derived from diffusion tensor imaging (e.g., *CLU*, Braskie et al., 2011; *BDNF*, Chiang et al., 2011a; *HFE*, Jahanshad et al., 2012). Furthermore, it will be interesting to study interactions between genes discovered through GWAS by considering the overall pathways or regulatory networks in which they act (Inkster et al., 2010; Potkin et al., 2010). Another type of genetic information, not considered here, is rare variants on the genome (Schork et al., 2009), or copy number variants, which may also be relevant in the determination of brain structure. Ongoing imaging studies are beginning to include proteomic and gene expression data, as well. Such studies may begin to integrate genetic information from different sources to probe the mechanisms of brain pathology and identify means to intervene and resist it.

## Conflict of Interest Statement

The authors declare that the research was conducted in the absence of any commercial or financial relationships that could be construed as a potential conflict of interest.

## Footnotes

^1^Apolipoprotein E

^2^Clusterin

^3^Phosphatidylinositol binding clathrin assembly protein

^4^Complement component (3b/4b) receptor 1

^5^GRB2-associated binding protein 2

^6^Golgi membrane protein 1

^7^Transient receptor potential cation channel, subfamily C, member 4 associated protein

^8^Lecithin retinol acyltransferase

^9^Spinocerebellar ataxia type 1 protein

^10^Cluster of differentiation 33

^11^Family with sequence similarity 113, member B

^12^http://adni.loni.ucla.edu

^13^Translocase of outer mitochondrial membrane 40 homolog

^14^Glutamate receptor, ionotropic, *N*-methyl *D*-aspartate 2B

^15^http://www.sph.umich.edu/csg/abecasis/MaCH/index.html

^16^http://pngu.mgh.harvard.edu/∼purcell/plink/

^17^http://cran.r-project.org

^18^http://www.alzgene.org/
